# Supplementary material for: Mapping the evolution of fertility support policies in China: A content and instrumental analysis
Source: PLoS One. 2025 Oct 9;20(10):e0332137. doi: 10.1371/journal.pone.0332137 (PMC12510515; doi:10.1371/journal.pone.0332137)
Supplement: S1 Appendix — (ZIP) [file pone.0332137.s001.zip › S1 Appendix. 226 original policy documents/110-人力资源和社会保障部办公厅关于妥善解决城镇居民生育医疗费用的通知(FBM-CLI-4-120363).doc]

人力资源和社会保障部办公厅关于妥善解决城镇居民生育医疗费用的通知

**发布部门：** [人力资源和社会保障部](https://www.pkulaw.com/law/chl?Aggs.IssueDepartment=60236) [机构沿革](javascript:void(0))

**发文字号：**人社厅发〔2009〕97号

**发布日期：**2009.07.31

**实施日期：**2009.07.31

**时效性：** [现行有效](https://www.pkulaw.com/law/chl?Aggs.TimelinessDic=01)

**效力级别：** [部门规范性文件](https://www.pkulaw.com/law/chl?Aggs.EffectivenessDic=XE0302)

**法规类别：** [公费医疗](https://www.pkulaw.com/law/chl?Aggs.Category=09620)

人力资源和社会保障部办公厅关于妥善解决城镇居民生育医疗费用的通知
（人社厅发[2009]97号）

各省、自治区、直辖市人力资源社会保障厅（局），新疆生产建设兵团人力资源社会保障局：
　　为提高城镇居民医疗保障水平，保障妇女生育基本医疗需求，现就城镇居民生育医疗费用有关问题通知如下：
　　**一、**各地要将城镇居民基本医疗保险参保人员住院分娩发生的符合规定的医疗费用纳入城镇居民基本医疗保险基金支付范围。开展门诊统筹的地区，可将参保居民符合规定的产前检查费用纳入基金支付范围。

　　**二、**城镇居民生育医疗服务管理，原则上参照城镇居民基本医疗保险有关规定执行。要综合考虑城镇居民生育的基本医疗需求和基金承受能力，合理确定医疗服务范围和标准。要完善医疗费结算办法，探索适合生育保障特点的结算方式，为参保人员提供方便快捷的服务，控制医疗费用不合理支出。

　　**三、**各地要从实际出发，统筹考虑城镇居民基本医疗保险和城镇职工生育保险制度的衔接，积极探索保障城镇居民生育相关费用的具体措施，妥善解决城镇居民生育医疗费用问题。

人力资源和社会保障部办公厅
二〇〇九年七月三十一日

© 北大法宝：（[www.pkulaw.com](https://www.pkulaw.com/)) 专业提供法律信息、法学知识和法律软件领域各类解决方案。北大法宝为您提供丰富的参考资料，正式引用法规条文时请与标准文本核对。
欢迎查看所有[产品和服务](https://www.pkulaw.com/helps/2.html)。法宝快讯：[如何快速找到您需要的检索结果？](https://www.pkulaw.com/helps/70.html)[法宝](https://www.pkulaw.com/helps/69.html)V6有何新特色？

扫描二维码阅读原文

**原文链接：**<https://www.pkulaw.com/chl/a29e0f1d80a8e296bdfb.html>
